# Patterns of risk exposure in first 1,000 days of life and health, behavior, and education-related problems at age 4.5: evidence from *Growing Up in New Zealand,* a longitudinal cohort study

**DOI:** 10.1186/s12887-021-02652-w

**Published:** 2021-06-17

**Authors:** Jan L. Wallander, Sarah Berry, Polly Atatoa Carr, Elizabeth R. Peterson, Karen E. Waldie, Emma Marks, Stephanie D’Souza, Susan M. B. Morton

**Affiliations:** 1 Psychological Sciences and Health Sciences Research Institute, University of California, 5400 North Lake Rd., Merced, CA 95343 USA; 2 Auckland Museum, Auckland, New Zealand; 3 National Institute of Demographic and Economic Analysis, University of Waikato, Hamilton, New Zealand; 4 Faculty of Science, School of Psychology, University of Auckland, Auckland, New Zealand; 5 School of Population Health and Centre for Longitudinal Research He Ara ki Mua, University of Auckland, Auckland, New Zealand; 6 COMPASS Research Centre, Faculty of Arts, University of Auckland, Auckland, New Zealand

**Keywords:** Early childhood; development; risk exposure, Health, Obesity, Injury, Behavior problems, School start

## Abstract

**Background:**

Children who are high priority candidates for early intervention need to be identified to reduce their risk for experiencing problems in development. Those exposed to multiple risk factors are more likely to exhibit problems in development than those exposed to a single or no risk factor. We examined the longitudinal associations between persistence and timing of exposure to cumulative risk (CR) on three occasions by age 2 and problems in development at age 4.5 in health, behavior, and education-related domains.

**Methods:**

Data are from *Growing Up in New Zealand* (NZ), a prospective longitudinal study of a birth cohort first assessed during their last trimester in 2009–10 and followed at ages 9 months and 2 and 4.5 years. All women with an expected delivery date in a 12-month period who resided within a defined region were invited to participate, with no additional eligibility criteria. Exposure was measured for 12 sociodemographic and maternal health risk factors at third trimester and ages 9 months and 2 years, from which developmental trajectories were constructed capturing persistence and timing of CR exposure. Ten developmental outcomes were measured at age 4.5 to classify problems in overall health status, obesity, and injuries; internalizing and externalizing behavior problems; and letter naming, counting forward and backward, and expectations for starting school and completing education.

**Results:**

Analyses of data from 6156 children (49% female, 33% Non-European ethnicity) who participated in the 4.5-age assessment uniformly showed associations between exposure to more than consistently zero CR across early development and higher prevalence of being classified with problems for 9 of 10 outcomes. Persistent exposure to a CR ≥ 4 was generally associated with a higher prevalence of problems for 7 of 10 outcomes, whereas the timing of first exposure to CR ≥ 4 showed a less consistent association with problem outcomes.

**Conclusions:**

These findings are concerning because over 50% of NZ children are exposed to at least one of these risk factors at some point in early development. Routine screening of most of these risk factors during pregnancy is feasible and can identify priority candidates for intervention.

## Background

Children exposed to multiple risk factors in the sociodemographic and parental health domains are much more likely to exhibit problems in health and development than those who are exposed to a single or no risk factor [1–5]. For example, although teen-age motherhood is considered an important risk factor for poor child development [6, 7], by itself it only identifies a small portion of children with early difficulties [8]. In comparison, when teenage motherhood is added to a combination of other risk factors (e.g., low maternal education, maternal depressive symptoms), identification of children at risk of poorer development improves substantially [8].

This argues for the utility of the cumulative risk (CR) model, which accounts solely for the number of risk factors to which a person is exposed rather than the intensity of or unique set of risk exposures [9]. Risk factors are defined dichotomously (e.g., motherhood at ≤ age 19 vs. > age 19) and then summed, ignoring the combination of risk factors [10, 11]. Indeed, the particular set of risk factors appear less important for developmental impact than the number of factors to which a child is exposed [9–11].

In addition to the consistent finding that cumulative, relative to single or no, risk exposures have worse consequences for children’s health and development [2, 3, 9, 10, 12], there are substantive reasons for the widespread use of the CR model. Children are typically faced with constellations of risks rather than an isolated instance because risk exposures often co-occur (e.g., single-parent family, low-income household, crowded residence, high crime neighborhood, low quality schools) [9, 13]. Furthermore, some of the developmental correlates of major sociodemographic factors, such as poverty, are explained, in part, by exposure to multiple risk factors [14]. Finally, CR exposure research and theory is important because the number of children confronting multiple risk factors is large and expanding around the world [15].

Yet, despite this understanding being identified at least 20 years ago [2], the CR approach has rarely been applied to illuminate problems in *early childhood* development, because most CR research has focused on school age [9]. We are aware of only one study [12] that has examined how different developmental trajectories of CR exposure occurring in the critical early childhood period, from conception to about 2 years of age [16–19], are associated with subsequent child outcomes. Examining the same sample using the same methods as in the present study, that prior study was focused on the single outcome of total behavior problems [12]. Children exposed to any more than a consistent level of zero risk factors in the first 1000 days of life had a higher likelihood of being reported with a clinical level of total behavior problems at 4.5 years [12]. Consistent exposure to four or more risk factors across this early period had the highest prevalence at 44% [12]. Stimulated by these alarming findings, the question remains whether developmental trajectories of CR exposure are associated more broadly beyond behavior problems, potentially affecting children’s health and development more generally. Moreover, little is known about whether the persistence in and timing of CR exposure during this early life period matter for development. Such findings can inform when and how early screening and interventions should occur.

The allostatic load model [20] highlights the cumulative impact on the body caused by repeated mobilizations of multiple physiological systems in response to risk exposure, and can illuminate how CR can cause developmental disturbances [21, 22]. Indeed, CR in school age children has been shown to predict allostatic load both concurrently [11] and prospectively [23]. More frequent and persistent risk exposure elevates stress and accelerates this impact [20–23]. Moreover, when allostatic load occurs repeatedly, the physiological response systems become recalibrated, remaining on alert and altering their sensitivity to stresses. As well, the malleability of these response systems is compromised, so that they become less proficient in returning to a resting state when the stress desists. Response capabilities are therefore diminished by exposure to CR [20–23]. Drawing from the allostatic model, therefore, we would expect that any CR, but especially persistent CR exposure as well as CR exposure occurring closer to the developmental outcomes of interest, would predict problems in those outcomes.

We examine here the prospective longitudinal associations between exposure across three times in early development to CR, accounting for a range of sociodemographic and maternal health risk factors, and problems in development right before the start of formal education at age 4.5 across health, behavior, and education-related domains. Based on the allostatic load model [20–22], we hypothesize that there will be a higher likelihood of problems across domains when: (1) there is exposure to high CR at any point in early development compared with not at all; (2) exposure to high CR occurs persistently in early development compared with less persistently, and (3) when the timing of first exposure to high CR occurs later in this period and closer to the developmental outcomes compared to earlier.

## Methods

*Growing Up in New Zealand* (GUiNZ) is a prospective longitudinal cohort study of New Zealand (NZ) children. Detailed description of study design, recruitment, and demographic composition can be found in other publications and at http://www.growingup.co.nz/en.html [24, 25]. Ministry of Health Northern Y Regional Ethics Committee (NTY/08/06/055) provides ethical approval.

### Participants

As detailed elsewhere [24, 25], a broad range of strategies were employed to attempt to provide information about the study to all pregnant women in the sampling frame, a contiguous geographical area containing about one third of the NZ birth population. Pregnant women with expected delivery dates between 25th April 2009 and 25th March 2010 were eligible. There were no other inclusion or exclusion criteria. During the enrollment period in their third trimester, 6822 mothers consented and participated in the first assessment of risk factors (see below). Assessments were completed with the mothers of 6476 (95% of enrolled) children at age 9 months and 6327 (92%) at age 2 years. At child age 4.5 years, 6156 (90%) children and their mothers completed assessments, which informed the health and developmental outcomes of interest in this study, constituting the analysis sample for the current study.

Based on data available from Statistics New Zealand, the demographic characteristics of the mothers in the cohort are comparable with those of all NZ parents on maternal age, ethnicity, parity and socioeconomic status [24]. Furthermore, the cohort showed generally close alignment to all NZ births between 2007 and 2010 on several birth characteristics and child sex [25]. Allowing for multiethnic identification, as standard in NZ, mothers indicated their children were European for 71%, Māori 24%, Pacific 20%, Asian 16%, and/or other ethnicities 3% First born comprised 42%. Mother’s educational attainment upon enrollment was 7.2% with no secondary school qualification, 23.9% secondary school qualification, 30.6% diploma or trade certificate, 22.6% Bachelor’s degree, and 15.6% above Bachelor’s degree. Additional demographics have been reported elsewhere [25].

### Procedures

Written informed consent was obtained from all participating mothers. Data collections administered by trained interviewers applied a variety of tools to obtain information on a broad range of developmental outcomes and potential determinants, mostly in face to face interviews with mothers [24]. All material was prepared in English. This was tested in a pre-sample of 200 families representing different ethnicities and language backgrounds. Then, at most five parents (< 0.1%) at each GUiNZ sample assessment requested the interview to be conducted in Te Reo Maori rather than English. This was accommodated using a translator throughout the complete interview. Children were directly assessed at age 4.5 years. The following risk exposure and problem outcome measures were relevant for this study:

### Risk exposures

*Risk factors* delineated in Table 1 were assessed at the antenatal (3rd trimester), 9-month, and 2-year assessments to comprise the CR index at each occasion [12]. Twelve sociodemographic and maternal health variables were identified a priori based on previous use in international studies as markers of disadvantage and vulnerability for poor outcomes in children, and to be (a) age and context appropriate, (b) available through routine data gathering, and (c) measurable in a standard manner [8, 12, 17]. Ten of the 12 risk factors were measured based on a question utilizing either single or few item responses from the mother, as noted in Table 1, leaving two exceptions:

**Table 1:** Definition and Prevalence of Risk Factors and Cumulative Risk Exposure and Categories

Risk factor	Definition	%		
		Antenatal	9 months	2 years
Maternal depression	EPDS ≥13, indicating likely depression	12	8	N/A
Maternal low health	Self-rated health = “fair” or “poor”	9	9	N/A
Maternal smoking	Smoke “regularly/every day”	10	13	13
Maternal young age	Age ≤ 19 at pregnancy	4	4	4
Maternal single status	No current partner	9	8	10
Maternal low education	No formal secondary school qualification	6	6	N/A
Maternal financial stress	Reporting “highly stressful” money problems	17	14	17
Maternal unemployment	Not on leave, actively seeking work, but not employed	7	6	7
Income tested benefit	Receiving income tested government benefit	14	17	16
Public housing residence	Residing in public/social housing	6	6	6
Overcrowded residence	≥ 2 persons per bedroom	12	20	19
Neighborhood deprivation	Residing in NZ Deprivation (2006) area deciles 9 or 10	25	24	24
**Cumulative risk exposure**				
0		46	43	41
1		23	24	24
2		12	12	13
3		7	8	8
4		4	5	7
5		3	2	3
6		2	2	2
≥ 7		2	1	< 1
**Cumulative risk category**				
Zero	0 risk factors	46	43	41
Medium	1–3 risk factors	42	44	45
High	≥ 4 risk factors	11	12	13

*N* = 6156. *N/A* not assessed, *EPDS* Edinburgh Postnatal Depression Scale, *NZ* New Zealand

Maternal depression was assessed with the Edinburgh Postnatal Depression Scale [26], a 10 item (each scored 0–3) self-report screening tool, focused on the cognitive and affective features of depression. A sum score cut-off ≥13 indicates significant depressive symptoms [26], providing satisfactory sensitivity (79%) and specificity (85%) for diagnosed clinical depression in non-postnatal women [27].

Neighborhood socioeconomic deprivation matched to the mother’s residential address was based on the NZ Deprivation 2006 Index [28], which is an area-level measure of relative population SES, determined using indicators from the 2006 NZ census. Scores range from least (decile 1) to most (decile 10) deprived. Here neighborhood deprivation was classified as High at deciles 9–10, which identifies the population living in the 20% most deprived neighborhoods.

*Development trajectories of CR exposure* were classified by counting the number of risk factors present separately at each of the antenatal, 9-month, and 2-year assessments, following previously established procedures [10]. Consistent with prior research [12, 29], exposure to a CR ≥ 4 was defined as High CR at each assessment. Exposure to CR = 0 was classified as Zero CR and a CR between 1 and 3 was classified as Medium CR. Applying the same procedure as in an earlier study [12], developmental trajectories of CR exposure could then be constructed by considering the CR classification across the three assessments. The focus in doing so was to capture different trajectories involving High CR at one or more assessments. As shown in Table 2, seven developmental trajectories, labelled #3 through #9, could be identified involving exposure to High CR (e.g., #4: Medium or Zero at antenatal/High at 9 mos./Medium or Zero at 2 yrs.; #9: High/High/High; #8: Medium or Zero/High/High). Trajectories of CR not involving any exposure to High CR were divided into two: One captured consistent Zero CR exposure (#1: Zero/Zero/Zero) across the three assessments, which served as the reference trajectory. The second (#2) included the remainder where there was no High CR exposure at any assessment (e.g., Medium or Zero/Zero/Zero; Medium or Zero/Medium or Zero/Medium or Zero) but excluded the consistent Zero CR (#1) trajectory. Thus, nine CR trajectories exhaustively classified all children (Table 2).

**Table 2:** Classification of Developmental Trajectories of Cumulative Risk (CR) Exposure

Developmental Trajectories of CR				Raw^**a**^	Imputed^**b**^
#	Antenatal CR	9 months CR	2 years CR	***n (***%)	***n (***%)
9	High	High	High	317 (5)	422 (7)
8	Medium or Zero	High	High	198 (3)	229 (4)
7	High	Medium or Zero	High	72 (1)	102 (2)
6	High	High	Medium or Zero	81 (1)	99 (2)
5	High	Medium or Zero	Medium or Zero	129 (2)	149 (2)
4	Medium or Zero	High	Medium or Zero	103 (2)	112 (2)
3	Medium or Zero	Medium or Zero	High	139 (2)	161 (3)
2	Medium or Zero	Medium or Zero	Medium or Zero	2037 (52)	2979 (48)
1	Zero	Zero	Zero	1761 (30)	1829 (30)

“Medium or Zero” indicates CR of 0–3 at this assessment but excludes those who consistently had CR = 0 exposure across all three assessments (who were classified as the #1 trajectory)

^a^*n* = 5837

^b^*n* = 6156

### Problem outcomes

Ten developmental outcomes in three domains were measured at 4.5 years of age, as summarized in Table 3. An outcome was classified as problematic based on a clinical definition when available. In the absence of such, an outcome was classified as a problem based on normative comparison, aiming generally to identify as close as possible 10% of the distribution with the worst outcome. The exception to the latter was the education-related performance outcomes, where a normatively low score was identified as falling as close as possible below the 25th percentile of the distribution, which was considered as placing the child at risk for difficulties upon school entry.

**Table 3:** Definition and Prevalence of Problem Outcomes at 4.5 Years of Age

Problem Outcome	Measurement	Definition of Problem Status	Raw %^**a**^	Imputed %^**b**^
*Health domain*				
Overall health status	Maternal rating on standard scale	Poor, Fair, or Good rating	14.3	14.5
Obesity	Measured child BMI	Standard age and gender clinical cut-offs	15.2	15.2
Injuries	Maternal report	≥ 2 requiring medical treatment since age 2	9.2	9.2
*Behavior domain*				
Internalizing problems	Maternal report on SDQ	Sum score ≥ 8 defined “clinical range”	10.6	10.6
Externalizing problems	Maternal report on SDQ	Sum score ≥ 10 defined “clinical range”	11.7	11.8
*Education-related domain*				
Letter naming	Child performance on DIBELS	≤ 1 correct	21.2	32.2
Counting forward	Child performance counting to 10	≤ 9 correct	21.6	22.2
Counting backward	Child performance counting from 10	= 0 correct	24.6	27.4
Starting school concerns	Maternal report on 5 items addressing concerns	Reporting high concerns relative to the norms	11.7	12.5
Educational completion expectation	Maternal report	Child expected not to advance past secondary school	12.5	11.7

*BMI* Body Mass Index, *SDQ* Strengths and Difficulties Questionnaire, *DIBELS* Dynamic Indicators of Basic Early Literacy Skills

^a^*n* = varying due to missing data, 5479–6156; ^b^ *n* = 6156

#### Health domain

Ov*erall health status* (OHS) was rated by the mother using the single item: “In general, would you say your child’s health is...” with a 5-point response scale (*excellent*, *very good*, *good*, *fair*, *poor*). Findings based on this item in numerous child health surveys have been consistent with theoretical expectations and support its validity as a measure of OHS [30–32]. At age 4.5, few children (14%) were rated with anything less than Very Good OHS (Excellent = 51%, Very Good = 35%). Consequently, aiming to identify children whose OHS may be of concern relative to normative standards at this age, the criterion ≤ Good rating was applied to classify children with reported OHS problem, which occurred for 14%. To examine the sensitivity of the results using this criterion, the more commonly used criterion, at least for older children, of being rated with an OHS ≤ Fair (= 3%) was applied. Repeating all analyses when applying this more stringent criterion for OHS problems (≤ Fair) produced effects sizes that were in the large majority of analyses higher than when applying the more liberal OHS criterion (≤ Good) chosen for this study (see Table 4). However, due to the low prevalence meeting the more stringent OHS criterion (3%), a higher number of non-significant findings resulted.

*Obesity* was classified by applying standard age and gender based clinical cut-offs to the Body Mass Index, which was calculated from trained interviewers measuring child height and weight without shoes, using a laser stadiometer and electronic digital scale according to standard protocols [33, 34]. Applying this criterion, 15% of the cohort was classified as obese at age 4.5.

*Injuries* since age 2, significant enough to require medical treatment by a doctor or dentist or in a health center or hospital, were reported by the mother. The criterion of ≥2 injuries, which applied to 9% of this cohort, was used to classify children with a high injury occurrence relative to normative standards at this age. To test the sensitivity of using this criterion, all analyses were repeated classifying children with any (≥ 1) injury since age 2. The identical pattern of results was obtained with highly similar effect sizes as for the criterion chosen for this study (Table 4).

#### Behavior domain

Behavior problems were measured with the parent-report Strengths and Difficulties Questionnaire for ages 4–16 [35], where 20 problem behaviors are rated on a three-point scale from *not true* (0) to *certainly true* (2). Psychometric studies with preschool children have shown satisfactory model fit, acceptable internal reliability, and results supporting its validity for screening for behavior disorders at this age [36–38]. According to the authors of this instrument, scores can be classified into a “clinical range” that is comprised as close as possible by 10% of the population [35], which was applied to two behavior problem sub-domains [36]: *Internalizing* problems were classified as a sum score ≥ 8 based on 10 emotional symptoms and peer problems scale items, which applied to 11% of this cohort, and *externalizing* problems as a sum score ≥ 10 based on 10 conduct and hyperactivity scale items, which applied to 12%.

#### Education-related domain

*Letter naming* was tested using the Dynamic Indicators of Basic Early Literacy Skills*®* (DIBELS) [39] Letter Naming Fluency test where a page of randomly presented letters was shown to the child, who was asked to name as many as possible in 1 min. The DIBELS has been validated in a NZ longitudinal sample across elementary/primary school [40]. Designed to test children ages 4.5–6, the criterion of ≤1 correctly named letter was applied, which classified 32% of this cohort with a low score [40, 41].

*Counting forward* was tested by asking the child to count up from 1 to 10. The criterion of ≤9 correct was applied, which classified 22% of this cohort with a low score.

*Counting backward* was tested by asking the child to count down from 10 to 1. The criterion of 0 correct was applied, which classified 27% of this cohort with a low score.

*Starting school concerns* were measured with five items rated by the mother regarding the child starting school (e.g., “… worried that [child] will find being apart from me too difficult”) using a five-point Likert scale (*strongly agree* to *strongly disagree)*. Without an obvious or previously applied cut-off available, we aimed to identify a group with unusually high concerns in comparison to the large majority of mothers. Based on a sum score, 12% of the distribution in this cohort was classified as mother reporting high concerns about the child starting school relative to the normative standards.

*Expecting low education completion* was measured with a single item asking the mother “how far in school … or higher education do you expect [child] to go?” Low expected completion was classified if the child was not expected to advance past secondary school completion, which applied to 12% of this cohort.

### Statistical analysis

All analyses were performed using IBM SPSS Statistics v26. Analyses were completed on children who participated in any degree at the 4.5-year assessment, when data on the 10 health and developmental outcome variables were obtained. Missing data on outcome variables in this assessment ranged between 0 to 11.0%, with four of these 10 variables yielding missing for > 5% of children (Obesity, Letter Naming, Counting Forward, Counting Backward). Moreover, a risk trajectory could not be classified for 5.2% of the children due to missing data. Because data on most of these variables were considered as Missing Not At Random [42], multiple imputation was applied to the data to account for uncertainty due to missing data and to reduce inclusion bias. An iterative Markov chain Monte Carlo method, with a maximum of 10 iterations, produced five sets of complete data with imputed values for the missing values. The following analysis then was applied to the pooled imputed data from this procedure.

Multivariable logistic regressions provided the odds of a significantly different prevalence associated with the eight different groups, each defined by a different CR trajectory, being simultaneously compared with the stable Zero CR (#1) trajectory group as the reference, separately for each outcome. No other covariates were included in the models. Odds ratios (OR) with 95th percentile confidence interval (CI) were examined. Hypotheses 1–3 were tested by comparing certain CR trajectories representing different experiences of persistence and timing of CR exposure, as detailed in the Results.

## Results

### Longitudinal attrition analysis

At age 4.5, 6156 of the GUiNZ child cohort were assessed. Compared to those who completed the assessment at age 4.5 years, those cohort members who had been enrolled in GUiNZ but who did not complete this assessment, were over-represented with mothers who were teenagers when the child was born (17% vs. 37%), did not reside with a co-parent (33% vs. 51%), reported the child to have a non-European ethnicity (42% vs. 78%), and resided in neighborhoods with high deprivation (36% vs. 59%) [43].

### Prevalence of risk exposure and CR trajectories

The prevalence of each risk factor at each assessment is reported in Table 1. The most common risk factors were neighborhood deprivation (24–25% across assessments), maternal financial stress (14–17%), and income tested benefit (14–17%), as well as overcrowded residence after the child was born (19–20%). As also detailed in Table 1, exposure to High CR on at least one assessment occurred for between 11 and 13% of the cohort, with the remaining portion about evenly divided between being exposed to Medium (42–45%) or Zero (41–46%) CR. As specified in Table 2, consistent exposure to High CR across the three assessments (trajectory #9) occurred for 7%. Various combinations of exposure to High CR on one or two assessments (#3 - #8), but not three, occurred for 2–4% of the cohort, whereas consistent exposure to Zero CR (#0) across the three assessments occurred for 30%.

### Prevalence of problem outcome

As shown in Table 3, applying the criteria described above resulted in classification of problems for between 9 and 15% of children for seven of the 10 outcomes. For the three education-related performance tasks, between 22 and 32% obtained a score defined as low.

### High CR exposure and problem outcomes in health domain (H1)

As shown in Table 4, generally children with any exposure to High CR at any time in early development (#s 3–7) had a higher likelihood of receiving low OHS ratings, from about two- to over three-times the odds (prevalence = 17–36%), compared to children with stable zero CR exposure (#1, 10%). Two exceptions were children exposed to the trajectory of High/Medium or Zero/High CR (#7, 13%) and Medium or Zero/High/Medium or Zero (#4, 8%). Even children exposed to a at least one occurrence of Medium CR level but no High CR (#2), had a 67% elevated likelihood of receiving low OHS ratings (15%) compared to those with consistent Zero CR (#1, 10%)

**Table 4:** Logistic Regression of Problem Outcomes at 4.5 Years of Age on Trajectories of Cumulative Risk Exposure in Early Development

					Health Domain									Behavior Domain						Education-related Domain														
Developmental Trajectories of CR					OHS ≤ Good			Obesity			Injuries ≥ 2			Internalizing Problems Clinical Level			Externalizing Problems Clinical Level			Letter Naming Low Score			Counting Forward Low Score			Counting Backward Low Score			Starting School Concerns High			Educational Completion Expectation Low		
#	Antenatal CR	9 months CR	2 years CR	***n*** (%)	%	OR^**a**^	(95% CI)	%	OR ^**a**^	(95% CI)	%	OR ^**a**^	(95% CI)	%	OR^**a**^	(95% CI)	%	OR^**a**^	(95% CI)	%	OR^**a**^	(95% CI)	%	OR^**a**^	(95% CI)	%	OR^**a**^	(95% CI)	%	OR^**a**^	(95% CI)	%	OR^**a**^	(95% CI)
9	High	High	High	422 (7)	35.6	**2.62**	1.87,3.67)	27.7	**4.33**	(3.27,5.73)	11.0	1.21	(0.83,1.77)	30.6	**11.97**	(6.22,23.04)	27.2	**6.19**	(4.37,8.77)	60.4	**5.76**	(4.16,7.96)	44.5	**5.66**	(4.50,7.10)	41.0	**2.80**	(2.05,3.84)	24.9	**3.71**	(2.16,6.37)	24.9	**3.33**	(1.64,3.30)
8	Medium or Zero	High	High	229 (4)	26.6	**3.48**	(2.41,5.02	28.7	**4.51**	(2.99,6.80)	8.3	0.90	(0.49,1.65)	26.7	**9.96**	(5.57,17.80)	25.7	**5.77**	(3.94,8.46)	51.4	**4.06**	(2.53,6.50)	33.3	**3.55**	(2.59,4.87)	38.4	*2.55*	(1.58,4.11)	17.7	**2.44**	(1.59,3.75)	17.7	1.53	(0.81,2.90)
7	High	Medium or Zero	High	102 (2)	12.9	1.40	(0.67,2.93)	28.3	*4.63*	(2.08,10.27)	8.8	0.96	(0.43,2.16)	22.7	*8.19*	(2.06.32.48)	20.9	**4.55**	(2.24,9.24)	50.4	**3.92**	(2.37,6.48)	34.8	**2.23**	(1.51,3.29)	38.9	*2.58*	1.37,4.84)	18.4	**2.61**	(0.86,7.75)	18.4	*2.69*	(1.16,6.26)
6	High	High	Medium or Zero	99 (2)	17.2	*1.99*	(1.04,3.80)	26.3	**4.02**	(2.28,7.09)	6.5	0.68	(0.30,1.59)	23.8	*8.36*	(2.66,26.35)	16.0	*3.16*	(1.48,6.73)	57.0	**5.05**	(3.01,8.47)	44.2	**6.28**	(4.19,9.41)	47.7	**3.63**	(2.02,6.55)	21.4	*3.04*	(1.65,5.60)	21.4	*2.34*	(1.24,4.41)
5	High	Medium or Zero	Medium or Zero	149 (2)	17.2	*1.99*	(1.25,3.18)	20.3	**4.91**	(3.24,7.45)	9.7	1.05	(0.59,1.90)	18.2	**5.98**	(2.73,13.13	19.7	**4.03**	(2.46,6.73)	48.1	**3.50**	(2.38,5.14)	43.0	**4.60**	(3.17,6.66)	38.6	**2.53**	(1.75,3.66)	16.7	*2.24*	(1.35,3.72)	16.7	*1.89*	(1.11,3.22)
4	Medium or Zero	High	Medium or Zero	112 (2)	8.1	1.83	(0.89,3.74)	18.5	*2.54*	(1.36,4.73)	10.9	1.20	(0.64,2.24)	17.5	**5.72**	(2.70,12.13)	17.6	**3.55**	(2.04,6.20)	50.1	**3.78**	(2.37,6.02)	39.4	**4.22**	(2.82,6.31)	44.6	*3.24*	(1.70,6.19)	18.9	*2.60*	(1.42,4.78)	18.9	*2.46*	(1.30,4.65)
3	Medium or Zero	Medium or Zero	High	161 (3)	22.3	**2.79**	(1.66,4.68)	23.0	**3.35**	(2.15,5.21)	9.3	1.00	(0.56,1.77)	13.3	**4.07**	(2.03,8.16)	17.4	*3.56*	(1.71,7.43)	50.5	**3.87**	(2.67,5.62)	26.7	**2.09**	(1.39,3.15)	38.8	**2.57**	(1.75,3.77)	12.8	1.62	(0.94,2.81)	16.7	*2.16*	(1.24,3.75)
2^b^	Medium or Zero	Medium or Zero	Medium or Zero	2979 (48)	14.9	**1.67**	(1.38,2.03)	14.5	**1.90**	(1.52,2.40)	9.0	0.97	(0.79,1.19)	9.3	**2.76**	(1.56,4.88)	11.0	**2.04**	(1.60,2.60)	29.8	**1.60**	(1.37,1.87)	20.1	**1.62**	(1.38,1.90)	26.3	**1.43**	(1.21,1.71)	12.1	*1.53*	(1.19,1.99)	11.5	*1.37*	(1.10,1.70)
1	Zero	Zero	Zero	1829 (30)	9.5	REF		8.2	REF		9.9	REF		3.6	REF		5.7	REF		21.1	REF		14.1	REF		19.9	REF		8.2	REF		8.6	REF	
	Total sample			6156	14.3			15.2			9.2			10.6			11.8			32.3			22.2			27.4			11.7			12.5		

All analysis on multiple imputed data. *CR* cumulative risk, *OHS* Overall health status, *BMI* Body Mass Index, *CI* confidence interval; High = CR ≥ 4; Medium = 1 ≤ CR ≤ 3; Zero = CR = 0; REF = reference category

^a^**bold** indicates significant OR at *p* < .001 and *italics* indicate significant OR at *p* = .05 when compared with consistent Zero CR (#1) exposure trajectory

^b^This trajectory includes those with Medium or Zero CR exposure at each assessment but excludes those who consistently had CR = 0 exposure across *all* 3 assessments, who were instead classified as the #1 trajectory

.

Likewise, children with exposure to High CR at any time (#s 3–9) had a higher likelihood of being classified with obesity (19–29%) compared to those with consistent Zero CR exposure (#1, 8%). The odds of obesity for those with High CR at any assessment was between 2.5 and 4.5 times higher compared to those with consistent Zero CR exposure (#1). Exposure to at least one occurrence of Medium but no High CR (#2, 15%) was associated with 1.9 times the likelihood of being classified with obesity. However, there was no association between any CR exposure in early development and experiencing two or more injuries.

### High CR exposure and problem outcomes in behavior domain

As shown in Table 4, children with exposure to High CR at any time (#s 3–9) had a higher likelihood of being reported with problematic behavior in both the internalizing (13–31%) and externalizing (16–27%) domains compared to those with consistent Zero CR (#1, 6%) exposure, with odds from four to 12 times higher. For children exposed to a Medium CR level at least once (#2, 9–11%), the odds of classification with behavior problems in either domain were also higher compared to those without CR, with the odds of problematic behavior being two to 2.5 times higher compared to the reference group.

### High CR exposure and problem outcomes in the education-related domain

As detailed in Table 4, children with exposure to High CR at any time (#s 3–9) had a higher likelihood of scoring low on letter naming and counting forward and backward compared to those with consistent Zero CR (#1) exposure. This was also the case for children exposed to a Medium CR level at least once but no High level (#2). Odds of a low score on any of these performance tasks associated with any CR exposure were generally between at least two and over five times higher when compared to consistent Zero CR exposure (#1). Likewise, for children exposed to a Medium CR level at least once but no High (#2), the odds of obtaining a low score (20–30%) on any of these tasks were likewise higher, with about a 50% elevation of the odds, compared to the Zero CR reference group (14–21%).

Children with exposure to High CR at any time (#s 3–9) had a higher likelihood of mothers reporting high concerns about their starting school (13–25%) as well as expecting low educational completion (17–25%) compared to those with consistent Zero CR exposure (#1, 8–9%). Children exposed to a Medium CR level at least once but no High (#2), likewise had an elevated likelihood of mothers reporting low educational completion (11–12%).

### Persistence of high CR exposure (H2)

The effect of persistence in High CR exposure was tested in two ways. First, being exposed to High CR across all three assessments (#9) was compared to exposure to High CR at two of the three assessments (#s 6–8) across all problem outcomes. Results are detailed in the top panel of Table 5. Although results varied to some extent across problem outcomes and specific CR trajectories, generally the odds of being classified with a problematic outcome was significantly reduced when High CR occurred at only two compared to persisted across all three assessments for both types of behavioral problems, letter naming, counting forward, and maternal concerns about starting school. Prevalence of low OHS ratings was also reduced for two of the less persistent trajectories of High CR exposure (#s 6 and 7) but was increased for one (#8) compared to consistent exposure to High CR (#9) across the three assessments.

**Table 5:**
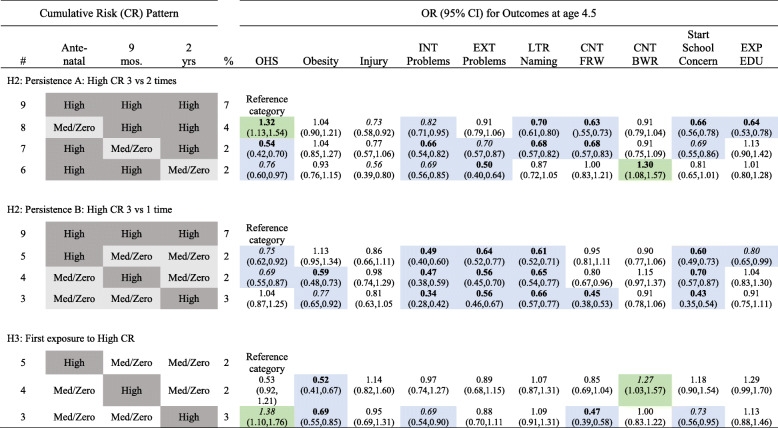
Comparison across problem behaviors for different patterns of persistence (H2) and timing (H3) of exposure to high cumulative risk

High = CR ≥ 4; Medium = 1 ≤ CR ≤ 3; Zero = CR = 0; *OHS* Overall Health Status, *INT* Internalizing, *EXT* Externalizing, *LTR* Letter, *CNT* Counting, *FRW* Forward, *BWR* Backward, *EXP EDU* Expected Education, *REF* reference category; *Med* Medium; **bold** indicates *p* <. 001*, italics* indicate *p* < .05; blue shading indicates significantly reduced odds, green shading indicates significantly increased odds compared to reference category

Second, being exposed to High CR across all three assessments (#9) was compared to exposure at only one of the assessments (#s 3–5). As detailed in Table 5, middle panel, only one exposure to High CR was generally associated with significantly reduced odds of classification with a problematic outcomes compared to persistent exposure across all three assessment. Generally, this odds reduction was between 40 and 60%. Exceptions were for injury and counting backwards, which showed no significant association with these CR exposure trajectories.

### Timing of high CR exposure (H3)

Comparisons of CR patterns reflecting the timing (Antenatal [#5] vs either at 9 months [#4] or 2 years [#3] of age) of a single exposure to High CR are detailed in Table 5, bottom panel. Associations were generally not consistently significant. The exception was for obesity, where a single exposure to High CR after birth (#s 3 and 4) was associated with approximately a 30–50% reduced likelihood of being obese compared to exposure only at the Antenatal assessment (#5).

## Discussion

Using longitudinal data that prospectively followed over 5800 children from the late antenatal period until 4.5 years of age, we examined how different patterns of timing and persistence of exposure to a high level (four or more) of sociodemographic and maternal health risk factors in early childhood were associated with problems across health, behavior, and education-related developmental domains. Results indicate that exposure to more than consistently zero risk factors, compared to those who consistently experienced zero risk factors over the early childhood period, is associated with a significantly higher likelihood of experiencing problems shortly before the start of elementary/primary school across nine of 10 outcomes, These outcomes included overall health, obesity, internalizing and externalizing behavior problems, letter naming, counting forward and backward, and mother having concerns about the child starting school and expecting the child not to continuing past secondary education. Consistent exposure to a high level of risk factors in early development was generally associated with the highest prevalence of problems. The exception was injuries, for which there was no association with risk exposures. These findings were therefore generally consistent with Hypothesis 1.

Consistent with Hypothesis 2, the likelihood of experiencing problems in these domains was generally reduced if exposure occurred less persistently, that is on one or two rather than all three assessment periods (antenatal, 9 months, 2 years). Nonetheless, even exposure to high level of risk factors on only one occasion was associated with a significantly elevated likelihood of problem outcomes compared to consistent zero risk exposure. The timing of one dose of high level of risk exposure in early development, whether at antenatal, 9 months, or 2 years, did not generally matter. The exception was that children exposed to a high level of risk factors on one occasion after birth, compared to only in the antenatal period, experienced a reduced likelihood of obesity. Therefore, findings regarding timing of exposure were generally inconsistent with Hypothesis 3. Taking the results overall, it appears that the effect from any exposure to CR is more important than the timing of it. It may also be that timing effects required examining relatively small subsamples, resulting in reduced power to detect consistent differences.

These findings are consistent with those from the few previous studies that have examined CR exposure as early in development as here. We are only aware of three other comparable studies. Sameroff and colleagues reported over 20 years ago that as CR increased, social adjustment and intelligence test scores decreased for children at 4 years of age [2, 44]. Results more recently from the Avon Longitudinal Study showed that a markedly larger portion (49% vs. 6%) of children with poor development by age five could be identified based on a CR model considering six factors rather than based only on the single exposure to teenage motherhood [8]. Likewise, a prior study of CR with this same NZ sample using the same methods as used here showed a significantly elevated risk for the single outcome of total behavior problems at 4.5 years of age associated with CR that was not consistently at zero in early childhood [12]. Internalizing and externalizing behavior problems were not differentiated in that study.

The present study is the first providing evidence that CR exposure is associated with elevations in problem prevalence broadly across multiple developmental outcomes, comprising health, behavior, and education-related domains. The present study also expands on previous findings to show that, although problems are most prevalent for children consistently exposed to high CR in early development, they are elevated even when exposure to a moderate level of CR (1–3 risk factors; trajectory #2) occurs in early childhood rather than remaining consistently at zero. These findings are disquieting because, whereas only about 10% are exposed to a high level of risk (CR ≥ 4) at any time, over 50% of NZ children are exposed to at least one of these risk factors at some point during their early development. Consequently, about one-half of all children may have a significantly elevated likelihood of experiencing problems in health, behavior, and/or education-related domains already prior to their starting formal schooling. It will be important to examine whether this risk continues to manifest itself as the children develop. This will be possible as GUiNZ continues to assess the cohort [24].

The CR approach is distinguished from examining adverse childhood events (ACEs), which is a distinct separate area of research. ACEs focuses on dysfunctional family experiences in childhood, which are self-reported retrospectively usually in adulthood, and typically without consideration for when in development they occurred [45, 46] The risk factors considered in the CR index examined here cover a considerably broader range of maternal sociodemographic and health-related conditions, most of which do not indicate dysfunction per se. Moreover, they are measured concurrently in our methodology.

Although the focus in this study has been on problem outcomes associated with exposure to high risk, it should be noted that the majority of children exposed even to a consistent high level of risk do not evidence problems at age 4.5. Future research must identify promotive and protective factors that support resilience in those children despite their high risk exposure. For example, although consistent exposure to high CR in early childhood is associated with a six-fold increased likelihood of being reported with an abnormal level of externalizing problem behaviors, over 70% of the children with this identical high level of risk exposure are not. What is it about them and/or their context that may contribute to their resilience?

The cumulative risk index used here includes various types of conditions. Some would be difficult to improve directly, such as neighborhood deprivation, but other risk factors appear more readily modifiable. Routine screening is feasible during pregnancy for several of the risk factors considered here [8]. For example, smoking during pregnancy can be reduced through intervention. Moreover, establishing pathways for mothers to continue or return to completing their education could directly reduce risk exposure as well as cause secondary, but important positive effects. For example, increasing education completion likely leads to better job prospects, which can lead to higher income, which can lead to affording less crowded housing possibly in less deprived neighborhoods.

### Limitations

The CR approach has some shortcomings. The designation of each of the risk factors here is arbitrary. Likewise, although with precedent from previous research [12, 29], so is designating as “high” risk the presence of four or more risk factors. Furthermore, information on risk intensity is lost with the CR approach and the CR index is additive precluding the possibility of statistical interactions between risk factors [9]. The determination of risk exposure here relied on maternal self-report, except for neighborhood deprivation. However, all but two of those risk factors (maternal depression and health) required maternal report of objective conditions. It should be considered that reporting on subjective conditions can vary due to educational attainment and emotional state. Some problem outcomes were present in a small number of children in some of the least prevalent CR trajectories, resulting in wide confidence intervals and low power to detect significant associations.

The OR should not be interpreted to represent the risk of a problem outcome associated with a certain trajectory of CR as it is known to provide an overestimate of the risk when outcomes are more than rare (generally > 10%) [47]. This is the case especially for the education-related outcomes Even though GUiNZ has one of the highest retention rates among longitudinal birth cohort studies [48], the analysis sample lost to follow-up at age 4.5 reflects retention bias, as is typical. Because this group was over-represented by children with higher vulnerability (e.g., single parenting, deprived neighborhood), the associations reported here are likely underestimates of the true association between CR exposure and problem outcomes. Finally, these data were collected in NZ, which manifests a distinct social context such that generalizing to other contexts should be done cautiously.

## Conclusions

CR can provide a useful mechanism to understand vulnerable subgroups and enable early identification and effective early intervention and support. For example, children with teenage mothers are not necessarily vulnerable only because of young maternal age, but more likely because this often co-occurs with other risk factors, such as lower access to education and financial stress, resulting in the accumulation of risk exposures. Children confronted by multiple risk factors, that is accumulating high CR, should be priority candidates for early interventions, regardless of which risk factors are involved. Such interventions should address the range of risk factors that they experience in addition to supports and strengths available [2]. Because the co-occurrences of risk factors have been shown here to be associated with a significant impact already in early development, systematic multisector interventions will likely be required to be implemented as early as possible in childhood to enhance development for vulnerable children. As suggested by the results from this study, disruption of the persistent exposure to a high number of risk factors early in life should be associated with a reduction in problems outcomes by age 4.5. However, this needs to be tested in a randomized controlled trial. Improved development for vulnerable children may in turn contribute to the reduction of health and social inequities in society.

## Data Availability

The data and material used in this study are available on request from the *Growing Up in New Zealand* study (please see https://www.growingup.co.nz/using-data, contact dataaccess@growingup.co.nz]. The data are not publicly available due to containing information that could compromise research participant privacy and consent.

## Abbreviations

CR: Cumulative risk
GUiNZ: Growing Up in New Zealand
NZ: New Zealand
OHS: Overall health status
EPDS: Edinburgh Postnatal Depression Scale
DIBELS: Dynamic Indicators of Basic Early Literacy Skills*®*
